# A Meta-Analysis of the Effect of Sodium Glucose Cotransporter-2 Inhibitors on Metabolic Parameters in Patients With Polycystic Ovary Syndrome

**DOI:** 10.3389/fendo.2022.830401

**Published:** 2022-02-21

**Authors:** Binayak Sinha, Samit Ghosal

**Affiliations:** ^1^ Department of Endocrinology, AMRI Hospitals, Kolkata, India; ^2^ Department of Endocrinology, Nightingale Hospital, Kolkata, India

**Keywords:** PCOS (polycystic ovarian syndrome), metabolic dysfunction, hormonal dysfunction, SGLT-2i, meta- analyses

## Abstract

**Objective:**

Polycystic ovary syndrome (PCOS) is the most common endocrinopathy among women of childbearing age and is associated with multiple morbidities. However, treatment for this condition is mainly applied for symptomatic relief and does not address the complex pathophysiology of this condition. This meta-analysis was conducted on the usage of sodium-glucose cotransporter 2 inhibitors (SGLT-2is) in PCOS because this group of drugs presents an attractive strategy to address the metabolic and hormonal defects by managing the pathophysiological defects observed in this syndrome.

**Methods:**

We included prospective trials that enrolled patients with established PCOS and compared an SGLT-2i group versus a control group with at least 2 weeks of follow-up. The standardized mean difference (SMD) was used for effect size estimation from individual studies and was pooled using the fixed effect model.

**Results:**

We included four trials with a pooled population of 158 patients with documented PCOS who received either an SGLT-2i or standard management. From a metabolic perspective, significant improvements were observed in the reduction in body weight (SMD: -0.68, 95% CI -1.16 to -0.19, <0.01), fasting plasma glucose (FPG) (SMD: -0.59, 95% CI -0.99 to -0.19, P<0.01), and insulin resistance as assessed with the HOMA-IR (SMD: -0.39, 95% CI -0.76 to -0.03, P=0.03). In addition, a significant improvement was noted in dehydroepiandrosterone sulphate (DHEAS) levels (SMD: -0.55, 95% CI -0.94 to -0.16, P<0.01).

**Conclusion:**

SGLT-2i use is associated with salutary outcomes of metabolic and anthropometric markers of PCOS and likely favourable hormonal effects.

**Clinical Trial Registration:**

[https://www.crd.york.ac.uk/prospero/display_record.php?ID=CRD42021268564], PROSPERO 2021 CRD42021268564.

## Introduction

Polycystic ovary syndrome (PCOS) is the most common endocrine disorder in women, affecting nearly 3.4% of women worldwide (1). PCOS constitutes a heterogeneous group of endocrine disorders manifesting predominantly as hyperandrogenism (HA) and ovulatory dysfunction (OD) (2). A strong metabolic component is associated with PCOS in the form of obesity, insulin resistance, and associated cardiovascular risk factors (3). Although the aetiology is not precisely known, PCOS is most likely a polygenetic disorder with associated epigenetic and environmental factors contributing to hormonal and metabolic imbalances in the short term (4).

The trio of hyperandrogenism, hormonal imbalance, and metabolic dysregulation, predominantly in the form of insulin resistance (IR), is responsible for most of the clinical manifestations associated with this syndrome. In patients with an underlying genetic predisposition, the primary defect manifests as disruption at the level of the hypothalamus-pituitary-ovarian axis, resulting in an excess of luteinizing hormone (LH), which alters the LH:follicle stimulating hormone (FSH) ratio and consequently reduces the FSH level. An LH abundance leads to hyperstimulation of thecal cells and a resulting androgen excess, whereas reduced FSH levels and elevated anti-Mullerian hormone (AMH) levels lead to ovarian arrest and anovulation (5). Elevated androgen levels not only lead to associated clinical features manifested as hirsutism and acne but are also responsible for elevated insulin-like growth factor-1 (IGF-1) levels, leading to metabolic abnormalities (insulin resistance, dysglycaemia and dyslipidaemia). This hormonal and metabolic association results in a vicious cycle, with the former responsible for the latter. Additionally, metabolic disturbances in the form of insulin resistance raise both the growth hormone releasing hormone (GnRH) and LH levels, disrupting the hormonal milieu (6). In addition, IR reduces the sex hormone binding globulin (SHBG) level, resulting in excess availability of free androgens and consequent androgenic manifestations (7).

The diagnosis of PCOS is standardized using Rotterdam’s criteria (2003), which require two of the three manifestations (HA, OD, and polycystic ovarian morphology [PCOM]), and the Androgen Excess and PCOS society criteria (2006), requiring both HA and OA for diagnosis (8).

The management strategy of PCOS does not involve targeting the syndrome complex per se but addressing the individual components, depending upon the presenting clinical features and the associated hormonal imbalances. Traditionally, these individual manifestations are targeted using hormonal contraceptive agents to regularize menstrual abnormalities; clomiphene citrate, letrozole, and metformin (especially in those with dysglycaemia) to treat anovulation; and birth control pills, spironolactone, and Eflornithine cream for hirsutism (9).

However, a large gap remains in addressing metabolic abnormalities and their associated cardiovascular risk (CV). Newer antihyperglycaemic agents, such as glucagon-like-peptide 1 receptor agonists (GLP1-RAs) and sodium-glucose cotransporter 2 inhibitors (SGLT-2is), improve hyperglycaemia, reduce IR, and produce significant weight loss, presenting an attractive strategy capable of addressing the metabolic defects of PCOS. In addition, both GLP1-RAs and SGLT-2is are known to significantly prevent the progression from prediabetes to diabetes and reduce the risk for CV events (in both diabetic and nondiabetic populations) (10, 11).

This meta-analysis is aimed at assessing the utility of SGLT-2 is in the management of the hormonal and metabolic aspects of PCOS and was designed following the PICO question format (shown below):

P (patient population) = Patients diagnosed with polycystic ovary syndrome.

I (intervention) = Received drugs in the SGLT-2i group.

C (control group) = Compared to a control group that received a placebo or an active control arm.

O (outcome) = Outcomes of interest included clinically relevant metabolic and hormone parameters related to PCOS.

## Materials and Methods

This review adheres to the Preferred Reporting Items for Systematic Reviews and Meta-Analyses (PRISMA) statement (12). The trial was registered with PROSPERO (ID: PROSPERO 2021 CRD42021268564).

### Eligibility Criteria

Studies with a randomized prospective design recruiting women between 18 and 45 years of age with diagnosed PCOS, established either with the Rotterdam criteria or the Androgen Excess and PCOS society criteria, were eligible to be included in this meta-analysis. There was no cap on the number of patients recruited. Additional requirements included a minimum duration of follow-up of two weeks and studies reporting the standardized metabolic (body weight, total fat mass, HOMA-IR, fasting plasma glucose) and hormonal (FAI, TT, SHBG, DHEAS) outcomes.

### Search Strategy

**Figure 1:**
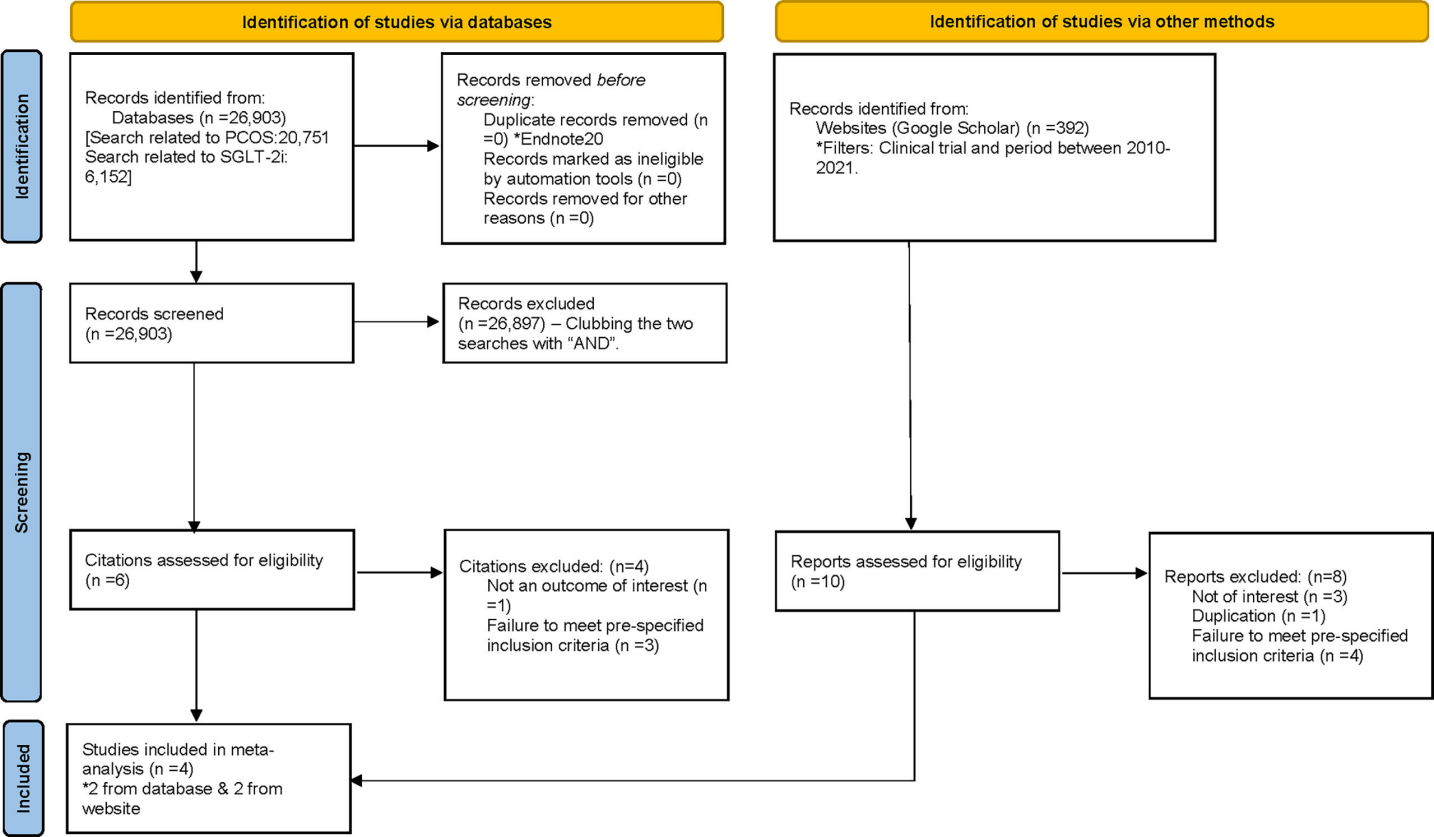
Study selection.

We performed a systematic review and meta-analysis. An electronic database search (PubMed) was performed using the two groups of terms (“Sodium-Glucose Transport Proteins”[Mesh] OR SGLT-2i*[tw] OR “sodium glucose co-transporter inhibitor*”[tw] OR Empagliflozin[tw] OR Dapagliflozin[tw] OR Canagliflozin[tw] OR Luseogliflozin[tw] OR Ertugliflozin[tw] OR Ipragliflozin[tw]) and (“Polycystic Ovary Syndrome”[Mesh] OR PCOS[tw] OR PCOD[tw] OR “polycystic ovary syndrome*”[tw] OR “polycystic ovarian syndrome*”[tw] OR “polycystic ovary disease*”[tw]). For the keywords nesting (“), truncation (*), and tagging ([tw]) were used to refine the search. The initial search was exported to EndNote version 20 to check for duplication. Combining the two group of searches using the Boolean “AND” along with applying an additional filter (clinical trial) yielded six citations (**Figure 1**). Applying additional eligibly filters based on pre-specified inclusion criteria and articles not of interest resulted in the selection of two citations (13, 14).

We included randomized, prospective studies that would automatically reduce the inherent biases. However, we were open to the control group in view of the paucity of data available in this field and included studies with a placebo as well as an active control arm.

In addition to the electronic database search, a manual search was conducted to identify any additional documents of interest. Two additional citations were identified by manual Google Scholar search (15, 16). Overall four citations were selected for analysis. The detailed search strategy is available in the Harvard Dataverse repository (**Supplementary Material**; 17).

### Study Selection

The authors independently performed the database search and assessed the studies for eligibility before sharing their respective outputs. All disputes were resolved by consensus.

### Quality Assessment

The risk of bias was assessed independently by both authors. The Cochrane risk of bias was used to assess the quality of the included studies. The individual components of the biases were assessed using the recommendations of Higgins and Altman (16). Any disagreement between the authors was resolved based on mutual consensus. There were no major issues identified in the studies by Javed et al. (13), Tan et al. (18), and Elkind-Hirsch et al. (14) (**Supplementary Material**; 17). Proper quality assessment could not be performed for the study by Cai et al. (15) since it was an abstract presented at the American Diabetes Association conference and was pending publication.

### Data Extraction

The electronic database search was conducted by both authors independently before comparing their results. A final joint database search was conducted before agreeing on the studies selected for further filtering. In the next step, filters (randomized trial and age range between 18 and 45 years) were employed to select studies of relevance. There were no restrictions regarding the language or date of publication. Any disagreement was resolved by consensus, and an additional search was conducted on a different day. After studies were identified to be included in the analysis, data were extracted from the studies including the trial name, author details, year of publication, place of origin of the study, mean age of participants, drugs used in the intervention and control arms, and all the prespecified outcomes of interest. The final step of data extraction depended on the inclusion criteria. Women with a differential diagnosis including Cushing’s disease, hyperprolactinaemia, 21-hydroxylase deficiency, or androgen secreting tumour; those who were planning to conceive or were pregnant; and those on medications with a potential to alter the outcomes of interest, such as hormonal contraceptives and steroids, were excluded from the analysis. The process of data extraction is detailed in **Figure 1**.

### Statistical Analysis

*A priori* power calculation based on a medium effect size, an alpha of 0.05 and a power of 0.80 was planned to determine the appropriate sample size for this meta-analysis. The G*Power 3.1.9.7 version was used to perform the power calculation.

In view of the extreme paucity of data on the subject under analysis, selection of the most appropriate method to report the effect size was a major challenge. Therefore, studies included in the analysis included both placebo and active comparator groups to increase the population and hence the reliability of the effect size. Because multiple arms were included in the study by Elkind-Hirsh et al. (14), we chose the exenatide QW arm as the comparator because of its metabolic noninferiority to dapagliflozin as established in the Duration 8 trial. We followed the method of the study by Javed et al. (13) where metformin was used in the comparator arm because any noninferiority of outcomes would indicate equal efficacy of SGLT-2is with the comparator. This was the basic logic for including both the active comparator and placebo in the control arm.

The analysis was performed using Comprehensive Meta-analysis software version 3 (Biostats Inc., Englewood, NJ, USA). The choice of selecting a fixed or random effect model was assessed using Cochran’s Q and Higgins’ I^2^ tests (an I^2^ ≥ 75% indicated considerable heterogeneity) and study characteristics (gross differences in estimated effect size as evident from differences in baseline characteristics).

A major obstacle to conducting an analysis using a uniform method was the different reporting techniques used in these studies. For example, Tan et al. (18) used the treatment ratio with significance levels to report outcomes, in contrast to Elkind-Hirsch et al. (14), where comparisons between individual arms were not reported. As a result, we used the standardized mean difference (SMD) as the effect size estimator, for which the significance is reported using a 95% confidence interval. Three methods were used to estimate the effect size: p-value for correlation, independent groups (mean, SD), and independent group (difference, p).

### Sensitivity and Subgroup Analysis

Because the control arm included both active controls (other antihyperglycemic agents) and nonactive controls (placebo), there is a risk of underestimating the effect size of the outcome of interest. To minimize this bias, a sensitivity analysis was undertaken by segregating the active-control and placebo groups (subgroups).

### Role of Funding

The authors did not receive any funding for this project.

## Results

### Search Results

As per the *a priori* power calculation a total sample size of 102 participants with 51 individuals in each group would result in an 80% power for estimating a moderate effect size in this meta-analysis (**Supplementary Material**; 17).

A detailed electronic database and manual search resulted in the selection of four studies (three full-text articles and one conference abstract) for inclusion in this meta-analysis.

A pooled population of 158 patients was analysed, with 78 individuals in the SGLT-2i arm and 80 individuals in the control arm. The baseline characteristics are detailed in **Table 1**. The age range was between 18 and 45 years, the baseline BMI was between 25 and 38 kg/m^2^, and the duration of follow-up ranged between 2 and 24 weeks. None of the patients had type 2 diabetes, but some did have insulin resistance of varying degrees as assessed by the baseline HOMA-IR.

**Table 1:** Baseline characteristics of the studies.

Study name (country/year)	Total number of patients (SGLT-2i/control)	Baseline BMI (kg/m^2^)	Baseline FPG (mg/dL)	Baseline HOMA-IR	Intervention arm (dose)	Control arm (dose)	Duration of follow-up (weeks)
Javed et al. (13) (UK/2019)	19/20	≥25	82.8 (median)	2.6 (median)	Empagliflozin (25 mg OD)	Metformin SR (1500 mg OD)	12
Tan et al. (18) (Germany & USA/2021)	15/14	38.1 (mean)	79.3 (median)	6.6 (median)	Licogliflozin (50 mg TID)	Placebo	2
Elkind-Hirsch et al. (14) (USA/2021)	17/20	>30 to <35	98 (mean)	4.1 (mean)	Dapagliflozin (10 mg OD)	Exenatide (2 mg) once weekly	24
Cai et al. (15) (China/2021)	27/26	NA	NA	5.4 (mean)	Canagliflozin (100 mg OD)	Metformin (1500–2000 mg -divided doses)	12

NA, Data not available.

The intervention arm received standard doses of SGLT-2is, while the control group varied according to the formulation of the drug used, except for the study by Tan et al. (18), where a placebo was used in the control arm. Javed et al. (13) used sustained release metformin, and hence, a once daily dose was given, in contrast to Cai et al. (15), where an immediate release metformin formulation was administered in divided doses (2–3 times daily). In the study by Elkind-Hirsch et al. (14), it was difficult to choose the control arm because it was a five-arm active comparator study. In view of the noninferiority data established in the Duration 8 trial between dapagliflozin and exenatide (once weekly) regarding metabolic parameters, we chose the latter as the active comparator arm (19). In addition, since both metformin and GLP1-RA are known to have beneficial effects in PCOS, any improvement in the prespecified outcome parameters or even equivalence would place SGLT-2is in a favourable position.

### Study Quality

The quality of these studies was assessed using the Cochrane risk of bias algorithm, and publication bias was assessed using funnel plots of the individual end points (**Supplementary Material**; 17).

### Metabolic Outcomes

**Figure 2:**
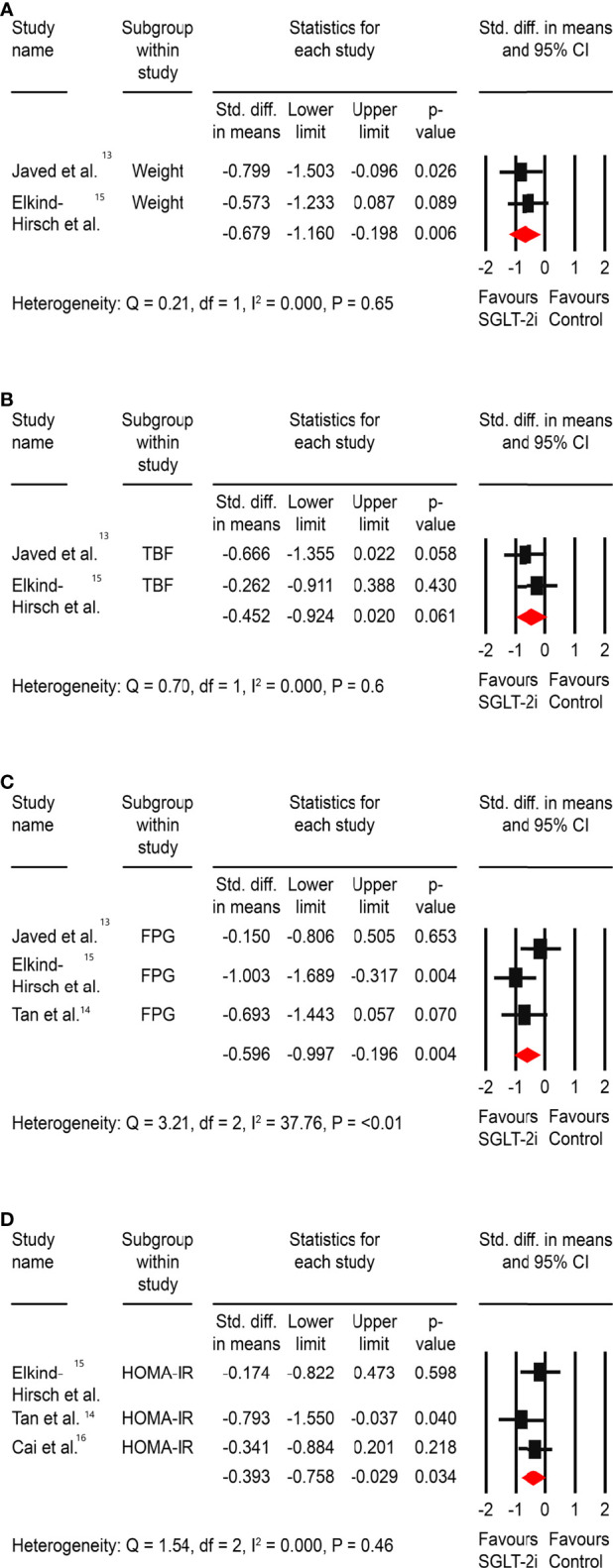
Effect of SGLT-2is versus control on **(A)** weight, **(B)** total body fat (TBF) %, **(C)** fasting plasma glucose (FPG), and **(D)** insulin resistance – HOMA-IR. *Std: Standard.

Significant improvements were observed in body weight (SMD: -0.68, 95% CI -1.16 to -0.19, <0.01), FPG (SMD: -0.59, 95% CI -0.99 to -0.19, P<0.01), and HOMA-IR (SMD: -0.39, 95% CI -0.76 to -0.03, P=0.03). However, there was no difference in the total body fat percentage change from baseline (SMD: -0.45, 95% CI -0.92 to -0.02, P=0.06) (**Figure 2**).

### Hormonal Outcomes

**Figure 3:**
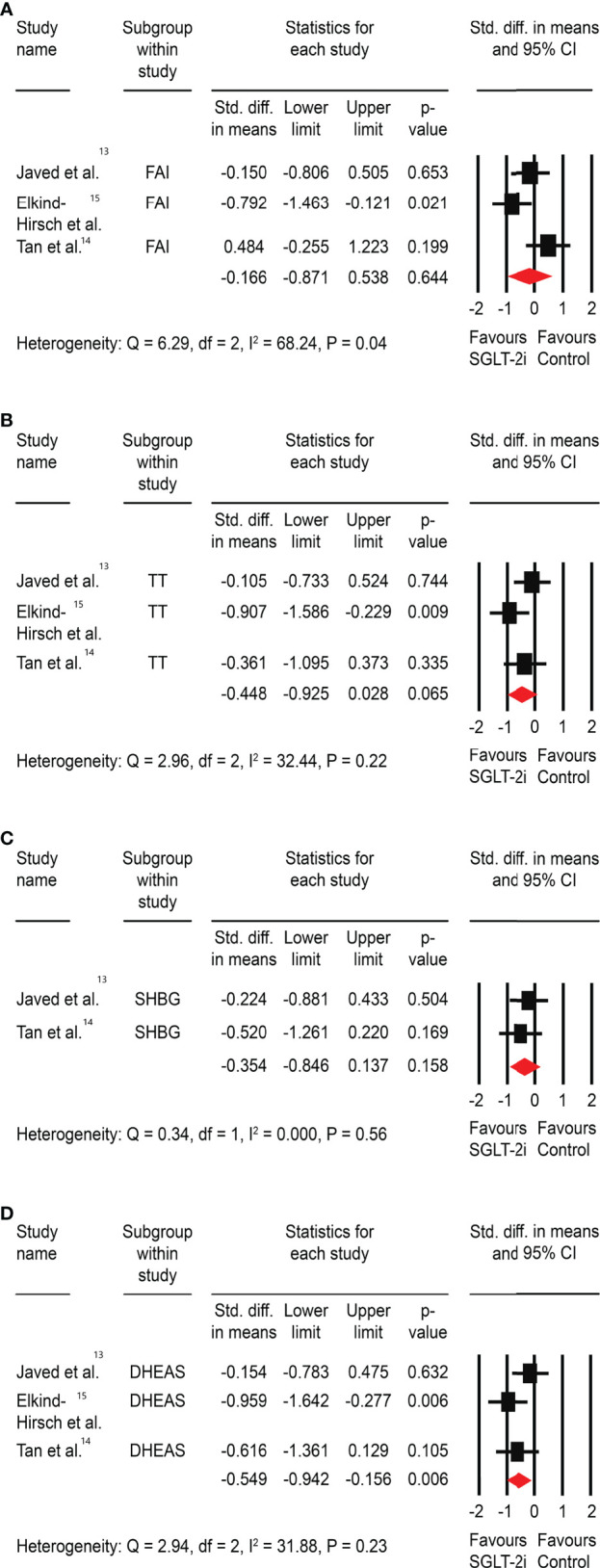
Effect of SGLT-2is versus control on **(A)** FAI, **(B)** total testosterone (TT), **(C)** sex-hormone binding globulin (SHBG), and **(D)** dehydroepiandrosterone sulphate (DHEAS). *Std: Standard.

There was no significant impact of SGLT-2is on FAI (SMD: -0.17, 95% CI -0.87 to 0.54, P=0.64), total testosterone (SMD: -0.45, 95% CI -0.93 to 0.03, P=0.07), or SHBG (SMD: -0.35, 95% CI -0.85 to 0.14, P=0.16). There was, however, a significant improvement in DHEAS (SMD: -0.55, 95% CI -0.94 to -0.16, P<0.01) (**Figure 3**).

### Sensitivity and Subgroup Analysis

Because of the absence of significant heterogeneity, we did not perform a sensitivity analysis. However, because the control arm included medications with proven metabolic and hormonal benefits for PCOS and only one study had a placebo in the comparator arm, we decided to conduct a subgroup analysis by excluding the study by Tan et al. (18). This could have blunted the significant metabolic and hormonal benefits expected with SGLT-2is, resulting in a comparative effect size. However, the metabolic benefit of FPG reduction was unaffected, the significant improvement in HOMA-IR disappeared, and the benefit on the hormonal parameter (DHEAS) was maintained (**Supplementary Material**; 17).

## Discussion

### Background Information

PCOS is a syndrome complex consisting of hormonal and metabolic disturbances and their associated clinical manifestations. The hallmark of hormonal imbalance is hyperandrogenism manifesting biochemically as an elevation of testosterone (T) and increased free androgen index (FAI), with the latter reflecting an increase in the T:SHBG ratio (20). Nearly 40%-70% of women with hyperandrogenism also exhibit an elevation of dehydroepiandrosterone sulphate (DHEAS) levels (21). The metabolic manifestations associated with PCOS include obesity, dysglycaemia, dyslipidaemia, sleep apnoea, and hypertension and are exhibited by nearly 33% of women (22). The biochemical hallmark of metabolic disturbances in PCOS is insulin resistance assessed with indices such as the homeostatic model assessment (HOMA) and quantitative insulin sensitivity check index (QUICKI), which have reasonable correlations among each other as well as with insulin clamp techniques (23).

Metabolic disturbances in PCOS are accompanied by heightened endothelial inflammation, leading to an increased risk of future CV events (24). According to a nationwide survey in Denmark, the hazard ratio for CVD was 1.7 in patients with PCOS, with obesity, dysglycaemia, hypertension, and dyslipidaemia being the principal contributors (25).

The traditional management strategies for PCOS predominantly target menstrual irregularities, hirsutism, and ovulation dysfunction. The only therapeutic strategy stressed from the metabolic perspective is weight reduction by lifestyle intervention. However, with the impressive results of GLP1-RAs and SGLT-2is on both the metabolic front and the associated CV outcomes, these molecules have come into focus in the management of a broad range of metabolic disorders, such as non-alcoholic fatty liver disease, sleep apnoea, and PCOS (26–28).

### Our Finding

**Figure 4:**
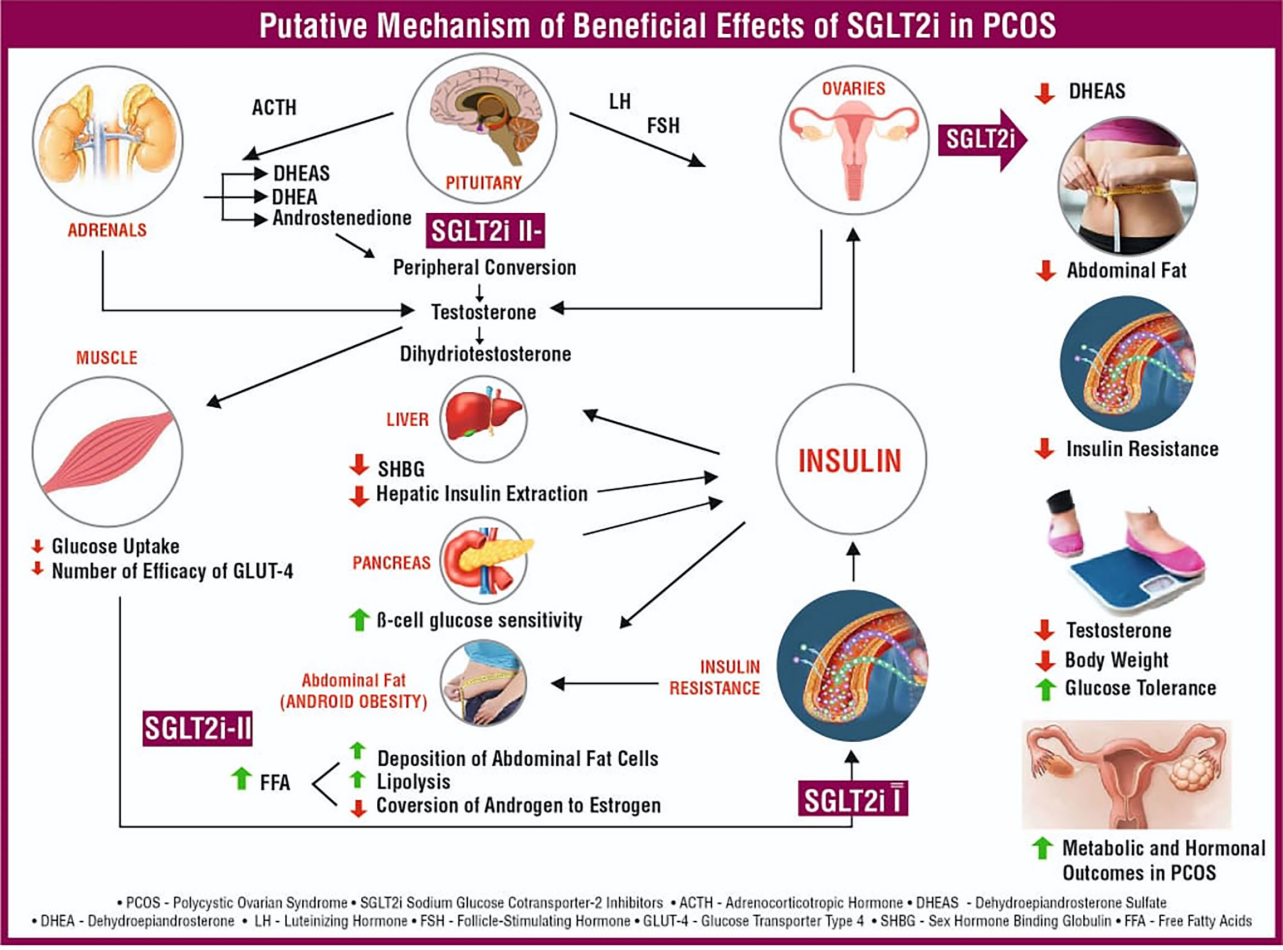
Putative mechanism of the beneficial effects of SGLT-2is in PCOS.

This meta-analysis was conducted to highlight the impact of SGLT-2is on metabolic and hormonal aspects of PCOS. To our knowledge, this is the first meta-analysis exploring these benefits. Significant improvements were observed in metabolic parameters, as demonstrated by reductions in body weight (SMD: -0.68, 95% CI -1.16 to -0.19, P<0.01) and FPG from baseline (SMD: -0.59, 95% CI -0.99 to -0.19, P<0.01) and improvement in the HOMA-IR (SMD: -0.39, 95% CI -0.76 to -0.03, P=0.03) with the use of SGLT-2is. No benefit on the HOMA-IR was found when the subgroup analysis was performed by excluding the placebo-controlled trial (18). One of the reasons for this finding could be the inclusion of active comparators such as metformin in the study by Javed et al. (13) and exenatide QW in the study by Elkind-Hirsch et al. (14), which could induce significant improvement in the HOMA-IR. Hence, it is highly plausible that the impact of SGLT-2is on the HOMA-IR was preserved in the subset of patients with PCOS, which is explained by their noninferiority to either metformin or exenatide QW. Regarding hormonal parameters, except for the impact on DHEAS (SMD: -0.55, 95% CI -0.94 to -0.16, P<0.01), which persisted even after the subgroup analysis, there was no significant difference observed for FAI, TT, and SHBG. The hyperinsulinaemia-induced elevation in dehydroepiandrosterone sulfate (DHEAS) could be an indicator of the risk of developing type 2 diabetes (T2D) in the future (29). A reduction in DHEAS levels, especially with increasing age, has been found to be protective against CV events (30). Moreover, the reduction in DHEAS shown here provides an interesting hypothesis wherein SGLT-2is, by their unique ability to reduce body weight and improve glucose uptake, would reduce hyperinsulinaemia, along with a reduced amount of DHEAS causing a reduction of free testosterone, which in turn would improve glucose utilization, forming a basis for breaking down the vicious cycle of hyperinsulinaemia and hyperandrogenism, the very basis of PCOS (**Figure 4**).

### Limitations and Strengths

All the studies were limited by small sample sizes, and each study investigated varying endpoints. Hence, the tested hypothesis needs to be analysed in a larger population of patients with a uniform and well-defined outcome criterion. Individual patient-related data were not available for analysis. We could not assess the extended metabolic parameters of interest, such as dyslipidaemia since data were either not presented or inadequately analysed in the studies. The association between body mass index (BMI) and PCOS is proven beyond doubt. Hence it would have been interesting to note the impact of different BMI subgroups on the outcomes associated with PCOS. This was another limitation of our analysis since only three of the four studies reported BMI and a subgroup analysis with such a small number was not possible. Finally, significant differences in the comparator arm could have skewed the data against the hypothesis.

Because of the very small number of patients recruited in the individual studies, as well as the urgent need to address the important aspect of metabolic management in PCOS, this meta-analysis is timely and pools an adequate number of patients to generate a robust hypothesis for future trials. None of the parameters exhibited significant heterogeneity, and hence, the effect size estimation was robust. The subgroup analysis was also supportive of the primary hypothesis.

### Literature Review

Empagliflozin was studied in 19 women with PCOS with metformin as a comparator and was found to improve body weight, basal metabolic rate, and parameters of glycaemia with a nonsignificant effect on androgens and other hormones (13). Dapagliflozin (DAPA) was studied alone and in combination with exenatide (EQW) and GLP1-RAs compared with a combination of phentermine/topiramate (PHEN/TPM). EQW/DAPA and PHEN/TPM resulted in the greatest loss of weight and total body fat according to DXA and WC. Despite equivalent reductions in BMI and WC with PHEN/TPM, only EQW/DAPA and EQW resulted in significant improvements in the mean blood glucose and insulin sensitivity (14). Reductions in fasting glucose, testosterone, FAI, and BP were observed with all drugs. In a randomized controlled trial of 53 patients in China, canagliflozin was found to be noninferior to metformin for all anthropometric, metabolic, and hormonal parameters (15). In another RCT, licogliflozin produced an improvement in metabolic parameters (18). In addition, licogliflozin usage was associated with a reduction in androstenedione and DHEAS, both precursors of testosterone, the driving force of hyperandrogenism in PCOS.

### Implications for Research and Practice

A definite need exists for treatment options to manage PCOS, a condition with far-reaching implications in both the short term and long term, caused by a deadly nexus of metabolic and hormonal defects. SGLT-2is, by their unique mode of action, powerful efficacy, and proven cardiovascular benefits, are naturally apt candidates (26). This meta-analysis indicates the benefits of using SGLT-2is for the treatment of PCOS and provides ample evidence to look forward to the publication of large-scale RCTs currently in progress (31).

## Conclusion

SGLT-2i use is likely to be associated with not only benefits on the anthropometric and metabolic outcomes of PCOS but also on the hormonal defects associated with this condition.

## Data Availability Statement

The original contributions presented in the study are included in the article/supplementary material. Further inquiries can be directed to the corresponding author.

## Ethics Statement

We dealt with published data and hence ethical approval was not taken.

## Author Contributions

The concept was conceptualized by BS. Electronic database search was conducted independently by BS and SG and the selected citations were identified based on consensus. The meta-analysis was conducted by SG. The manuscript was written by BS with contribution by SG in the methodology section. All authors contributed to the article and approved the submitted version.

## Conflict of Interest

The authors declare that the research was conducted in the absence of any commercial or financial relationships that could be construed as a potential conflict of interest.

## Publisher’s Note

All claims expressed in this article are solely those of the authors and do not necessarily represent those of their affiliated organizations, or those of the publisher, the editors and the reviewers. Any product that may be evaluated in this article, or claim that may be made by its manufacturer, is not guaranteed or endorsed by the publisher.

## Supplementary Material

All the supplementary materials are available from the Harvard Dataverse: https://dataverse.harvard.edu/dataset.xhtml?persistentId=doi:10.7910/DVN/PR3QSB
